# Intentional Carbofuran poisoning in 7 dogs

**DOI:** 10.1186/s12917-020-02534-w

**Published:** 2020-08-31

**Authors:** Dalma Pivariu, Adrian-Nechita Oros, Flaviu Tabaran, Adrian Gal, Cristian Martonos, Andras-Laszlo Nagy

**Affiliations:** 1 Department of Veterinary Toxicology, University of Agricultural Sciences and Veterinary Medicine, 3-5 Mănăştur Street, 400372 Cluj-Napoca, Romania; 2 Department of Veterinary Pathology, University of Agricultural Sciences and Veterinary Medicine, 3-5 Mănăştur Street, 400372 Cluj-Napoca, Romania; 3 Department of Veterinary Cell Biology, Histology and Embryology, University of Agricultural Sciences and Veterinary Medicine, 3-5 Mănăştur Street, 400372 Cluj-Napoca, Romania

**Keywords:** Carbamates, Furadan, Pathology, Veterinary forensic medicine

## Abstract

**Background:**

Carbofuran is a widely used broad-spectrum pesticide that, despite strict regulation and being banned for more than a decade, is still encountered in cases of intentional poisoning in dogs and wildlife. The objective of the study was to provide a complete and detailed description of the pathological, histological and toxicological findings of 7 cases of intentional carbofuran poisoning in dogs.

**Results:**

In this retrospective study, 7 cases of carbofuran intoxication recorded from July 2015 to June 2017 were analyzed. Following complete history recording, all cases were examined by complete necropsy and histopathology. Carbofuran intoxication was confirmed in all cases by gas chromatography. The postmortem examination revealed extensive hemorrhaging and congestion located mainly within the respiratory, nervous and cardiovascular systems, accompanied by degeneration and necrosis within the lungs, heart, and kidneys.

**Conclusions:**

Although carbamates have been banned in the European Union, carbamate poisoning is still frequently encountered, especially in wild animals. This paper will contribute to a better understanding of the occurrence and pathogenesis of acute carbofuran exposure in dogs and contribute some peculiar pathological features of this type of poisoning to the current literature.

## Background

Carbofuran (2,3-dihydro-2,2-dimethyl-7 benzofuranyl methyl carbamate) is one of the most frequently encountered carbamates. It was used in agriculture from the 1970s [1] until it was banned on 31.12.2009 in the USA [2] and 05.12.2011 in Romania by the Law of Chemical Substances nr 254.

Furadan can still be sourced from several countries, such as Tanzania and Uganda. A recent survey indicated that carbofuran was readily available in approximately 80% of the sampled agro-veterinary supply stores in Uganda [3].

The most commonly marketed carbofurans were furadan, Bay 70,143, Curater, D 1221, Yaltox, Furacarb, and ENT 27164 [4, 5]. Carbofuran is available in granular, liquid and powder formulations [5, 6] and was used in agriculture and forestry as a broad-spectrum systemic insecticide, nematicide and acaricide [3, 7].

Although this substance was banned almost 10 years ago, our cases show that it is still encountered in cases of intentional poisoning.

The main objective of this manuscript is to present the gross and histopathological findings of serial cases of carbofuran poisoning in dogs and the results of toxicological screening by GS-MS/MS of samples collected from deceased animals and to highlight the important contribution of such an analysis to criminal investigations.

Exposure to carbamates can occur by oral ingestion, inhalation or dermal absorption. Given the high toxicity of carbamates, any usage errors regarding mixing or storage of this substance can lead to intoxication.

The oral LD50 is approximately 3–19 mg/kg of body weight in different animal species [8]. The oral LD50 for dogs is 19 mg/kg, and the LC50 for inhalation is 52 mg/kg [9]. Young animals can be intoxicated by a lower dose than that required for adults because of their underdeveloped enzymatic system [10]. Repeated exposure, such as frequent spraying, can also cause intoxication. Animals can also be intoxicated by licking an empty container.

Carbofuran is metabolized into 3-hydroxycarbofuran and 3-cetocarbofuran, two highly toxic metabolites. The metabolism of carbamates in organisms is fast, and a large portion of metabolic activation and detoxification occurs in the liver. Excretion of metabolites occurs through the urinary and digestive tract and residues can be detected in feces, saliva and milk [11]. Vomit and/or diarrheic contents may be found near the animal in addition to remaining poisoned food [12, 13].

In animals, necropsies are performed by veterinary pathologists or veterinary general practitioners; these individuals should be made aware of the possible circumstances that led to the carbofuran-related death of the animal and follow established protocols, both for necropsy and sampling for toxicology. A toxicologist should always be consulted about the appropriate matrices according to the pathologist’s suspicions, as well as the best methods for handling the samples, to obtain consistent and reliable results [14–19].

The majority of animals fatally poisoned by carbamates usually present nonspecific gross and histopathological findings [20, 21], such as systemic congestion and multiple areas of hemorrhage [22–24].

## Results

The toxicological examination employing gas chromatography established the diagnosis; carbofuran was detected in all of the cases.

The pathological findings for each case are presented separately in Table 1, and we describe the most relevant gross and histopathological findings following carbofuran intoxication below.

**Table 1:** Postmortem necropsy findings

Case Nr	External	Musculo-scheletal	Gastrointestinal and liver	Cardiovascular	Respiratory	Urinary	Central nervous System
**I**	Pink colored foreign substance (abundant) staying the fur around the mouth and muzzle	No significant findings	Pink colored foreign substance (abundant) staying the GI content (mainly bread) and mucosa of the upper GI Diffuse, acute, minimal hepatic congestion	No significant findings	Congestion and acute pulmonary edema (diffuse, acute)	Renal severe congestion, cortical- tubular necrosis (bilateral, diffuse, severe)	Meningeal cerebral congestion (diffuse, mild)
**II**	Pink-coloring the fur around the muzzle and on parts of the fur with a pink substance Conjunctiva hemorrhage of the third eyelid, acute, severe bilateral hyphema	Thoracic-cervical muscular and subcutaneous hemorrhages, petechial and ecchymosis (associated with hemorrhagic lymph nodes) Hemorrhagic joint fluid	Pink colored foreign substance admixed with chicken meat, feathers and corn within the pharynx, esophagus and stomach Diffuse gastric and small intestinal congestion	Hemorrhagic pericardial content	Trachea: diffuse congestion (severe) Parietal sub pleural hemorrhages, Lung: Bilateral, multifocal-coalescing (ecchymosis) pulmonary acute hemorrhages, with diffuse congestion and edema (severe);	Renal Congestion; Urinary bladder: sub mucosal ecchymosis	Meningeal cerebral congestion and hemorrhage, diffuse, bilateral, acute, severe
**III**	Unilateral bulbar conjunctiva congestion	No significant findings	Pharynx diffuse congestion and edema Oral, esophageal and gastric contents with pink chicken carcass residue Focal small intestinal hemorrhages (trans mural)	Hemorrhagic pericardial content Aortic valvular diffuse edema	Larynx and trachea: diffuse congestion (severe) Acute, severe, bilateral pulmonary congestion and edema of the bronchi and trachea	Multifocal perivascular cortico-medullary hemorrhages	Meningeal cerebral congestion
**IV**	Pink coloring of the fur Severe, unilateral hyphema, Epistaxis	No significant findings	Pharyngeal, Esophageal and gastric contents of chicken carcass debris soaked in a pink substance Externally expressed lower digestive (colon) hemorrhages	No significant findings	Pulmonary edema	Sub mucosal hemorrhages (paint brush) Congestion	Congestion
**V**	Pink bright substance on the fur and muzzle Severe, acute, bilateral hyphema Epistaxis	No significant findings	Gastric pink food contenting the oral cavity on the esophageal level Gastro intestinal and splenic diffuse congestion	Pericardial hemorrhagic content	Acute, severe, bilateral pulmonary hemorrhages, confluent suffusions, edema in the main bronchi and trachea	Sub mucosal multifocal congestion (minimal)	Acute, bilateral, diffuse meningeal cerebral hemorrhage Diffuse brain edema
**VI**	Foamy salivation, epistaxis, oral cavity hemorrhages, diffuse uveal congestion and hyphema	Acute muscular hemorrhages, axillary area	Pink colored foreign substance (abundant) staying the gastric and duodenal content (bread) and mucosa	Subepicardial congestion, and sub endocardia hemorrhages (suffusions) (RV and A); aortic valvular diffuse edema	Trachea: diffuse congestion (severe) Lung: Multifocal-coalescing (ecchymosis) pulmonary acute hemorrhages, with diffuse congestion and edema (severe); diffuse alveolar emphysema	Congestion	Meningeal congestion (diffuse, mild)
**VII**	Epistaxis, diffuse uveal congestion and hyphema	Acute muscular hemorrhages thoracic-abdominal and lumbar (massive)	Gastric mucosa congestion (diffuse); gastric content-plastic (possible carbofuran container) admixed with meat (presumably bite), hair, grass and blood	Subepicardial hemorrhages (ecchymosis); Hemorrhagic pericardial content	Trachea: diffuse congestion (severe) Lung: Multifocal (ecchymosis) pulmonary acute hemorrhages diffuse congestion and edema (severe); focal emphysema	–	Meningeal congestion and edema (diffuse, severe)

**Fig. 1:**
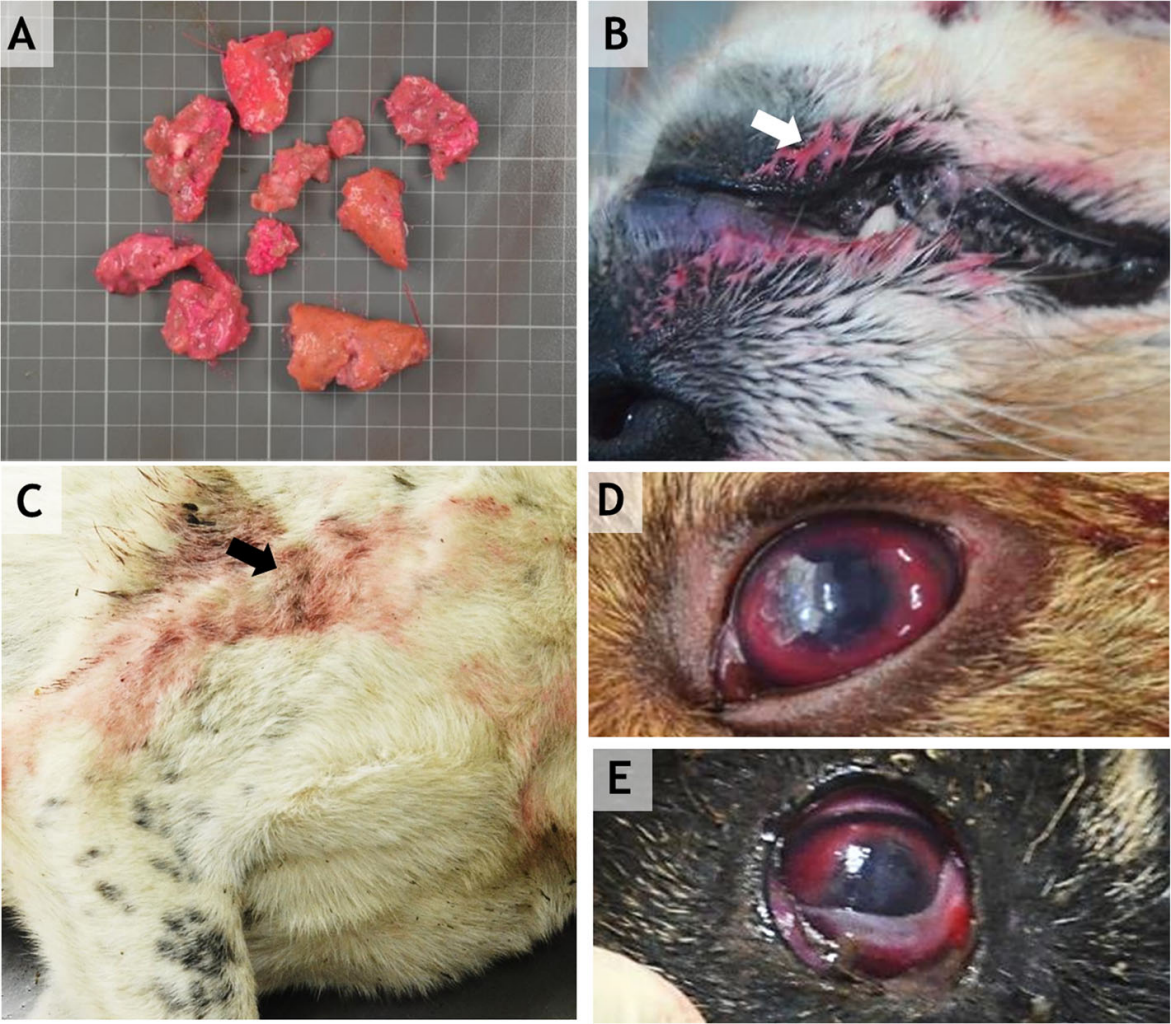
**a**: The bait showing abundant pink liquid. **b** and **c**: A pink liquid (presumptively furadan) staining the fur around the mouth and thorax. **d**: Diffuse uveal congestion and hyphema. **e**: Multifocal 3rd-eyelid hemorrhages

External examination of the body revealed dried saliva around the oral cavity and a pink color around the mouth (Fig. 1-b) and on some parts of the body, usually on the lateral parts where the animal may have touched its fur with its mouth (Fig. 1-c). In four dogs, we found epistaxis.

In the eyes, we noticed multifocal 3rd-eyelid hemorrhaging associated with diffuse uveal congestion and hyphema (unilateral or bilateral) (Fig. 1-d, e).

In three dogs, subcutaneous and muscular hemorrhages were present.

Generally, the gastric content was composed of an unknown pink-colored foreign substance mixed with bread or meat, chicken parts, or even feathers. In the pharynx and esophagus, pink coloration of the mucosa was noted. The small intestine and its content were also colored pink. Additionally, microhemorrhages were observed in the colon.

Histology revealed that the stomach mucosa had desquamation and catarrhal inflammatory lesions, mainly involving the superficial epithelium, and congestion in the deep part of the lamina propria and submucosa.

One dog presented diffuse, acute, and minimal hepatic congestion, and another dog had diffuse splenic congestion.

**Fig. 2:**
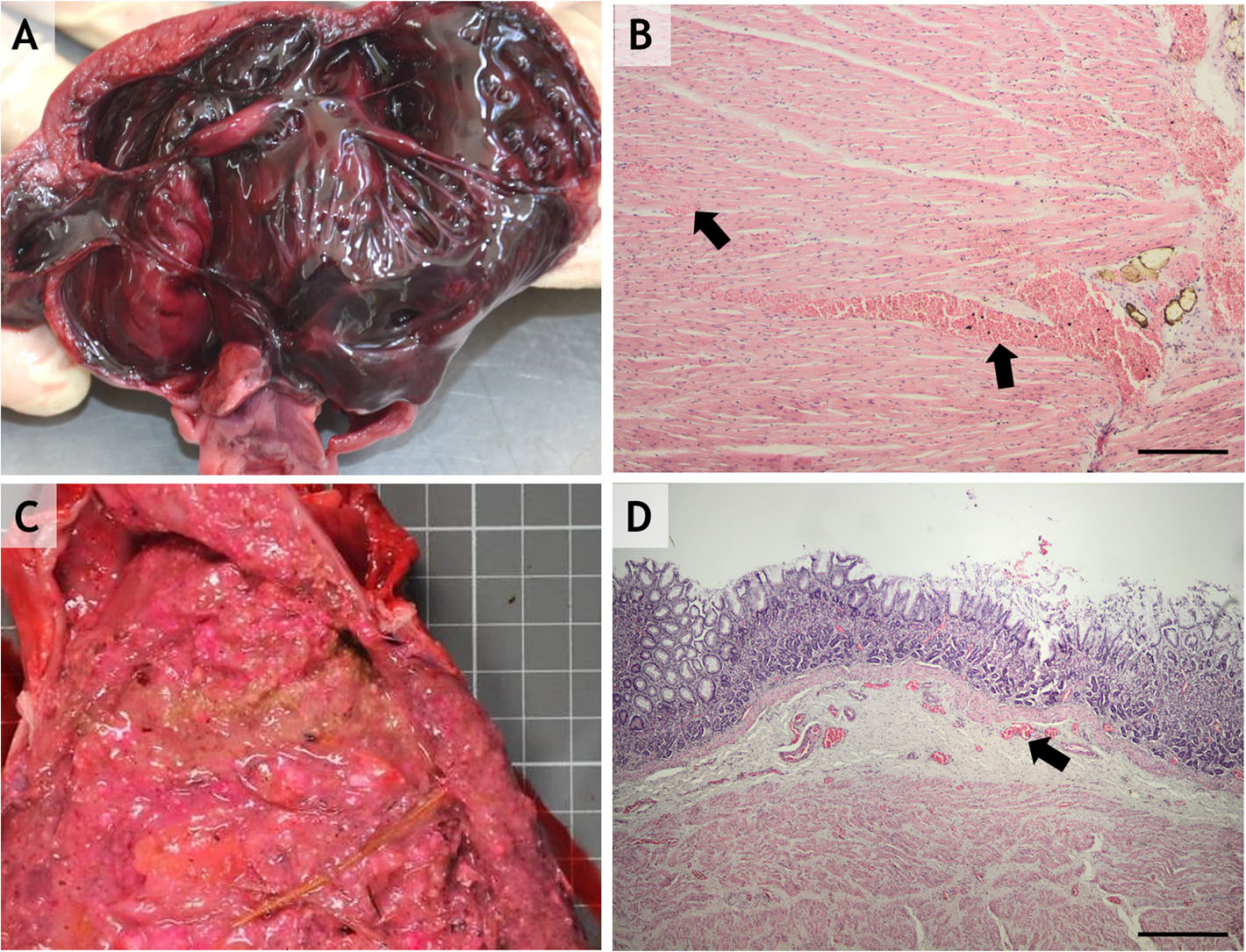
**a** Diffuse subendocardial hemorrhages. **b**: Myocardial congestion and hemorrhage H-E staining, 10x. **c**: Diffuse gastric congestion and gastric content containing a pink. Foreign substance. **d**: Diffuse gastric congestion within the lamina propria and submucosa; H-E staining, 10x; scale bar = 200 μm

Necropsy revealed hemorrhagic pericardial content and, in some cases, subendocardial congestion (Fig. 2a) and hemorrhage in the cardiovascular system. In the myocardium, diffuse congestion (Fig. 2-b) and hemorrhage were observed. In the myocardium of one particular individual, the myocardial fibers were replaced by fibrous tissue, with some atrophied myofibers sequestrated in the scar tissue, which could be the consequence of a chronic myocardial infarct.

**Fig. 3:**
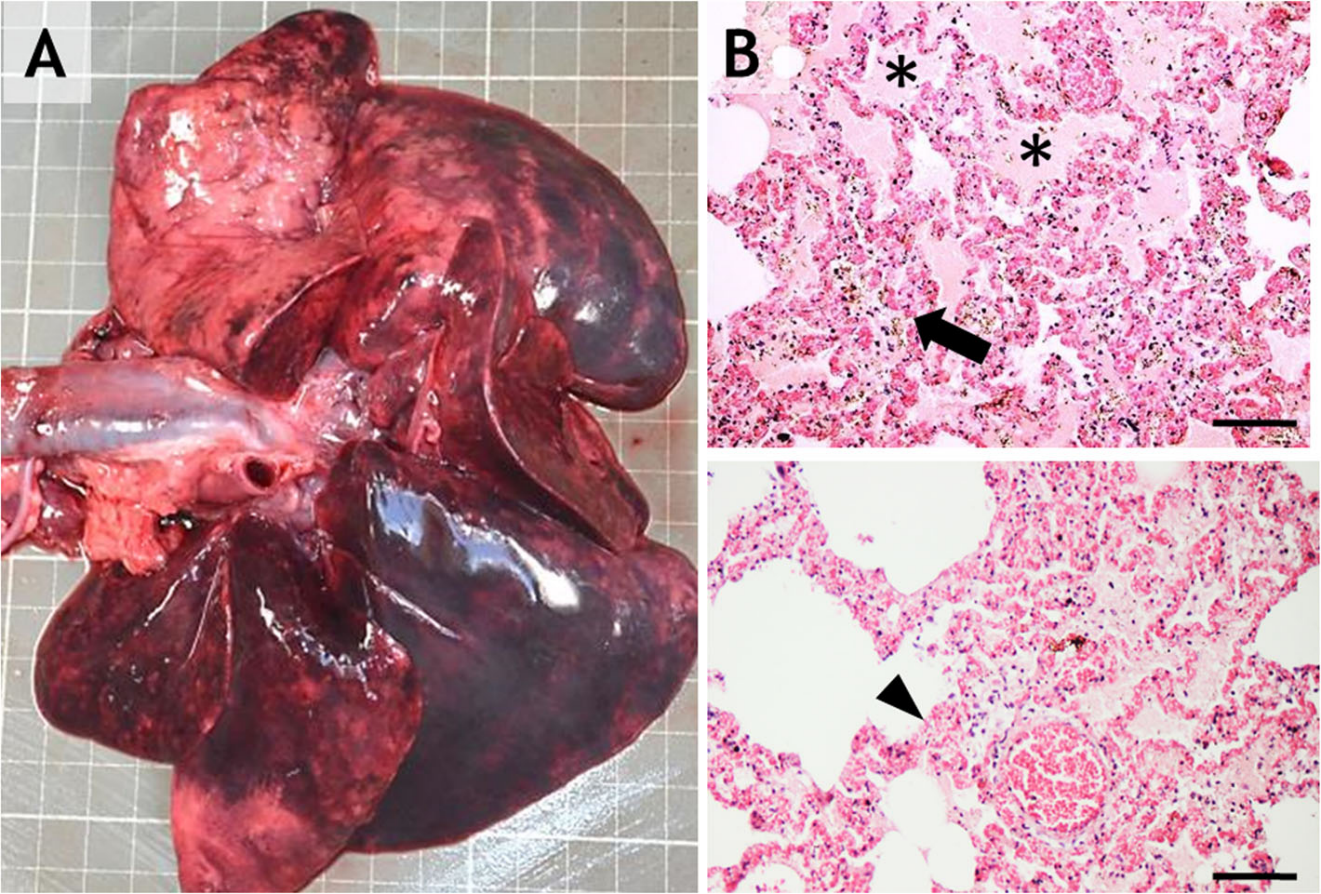
**a**: Acute, extensive and bilateral pulmonary congestion, edema, and multifocal hemorrhages. **b** and **c**: Alveolar-septal congestion (arrow), edema (asterisks) and hemorrhage (arrowhead); H-E staining, 20x; scale bar = 100 μm

In the upper respiratory tract, the larynx and trachea had diffuse congestion, and the lungs in most of the cases showed acute, severe, diffuse, bilateral pulmonary congestion and edema (Fig. 3–a), with multifocal petechial and ecchymosis.

Histologically, severe vascular changes, such as septal congestion (Fig. 3-c) associated with the presence of numerous siderocytes and diffuse edema in the alveoli, bronchioles and bronchi, were detected in the lungs (Fig. 3-b).

**Fig. 4:**
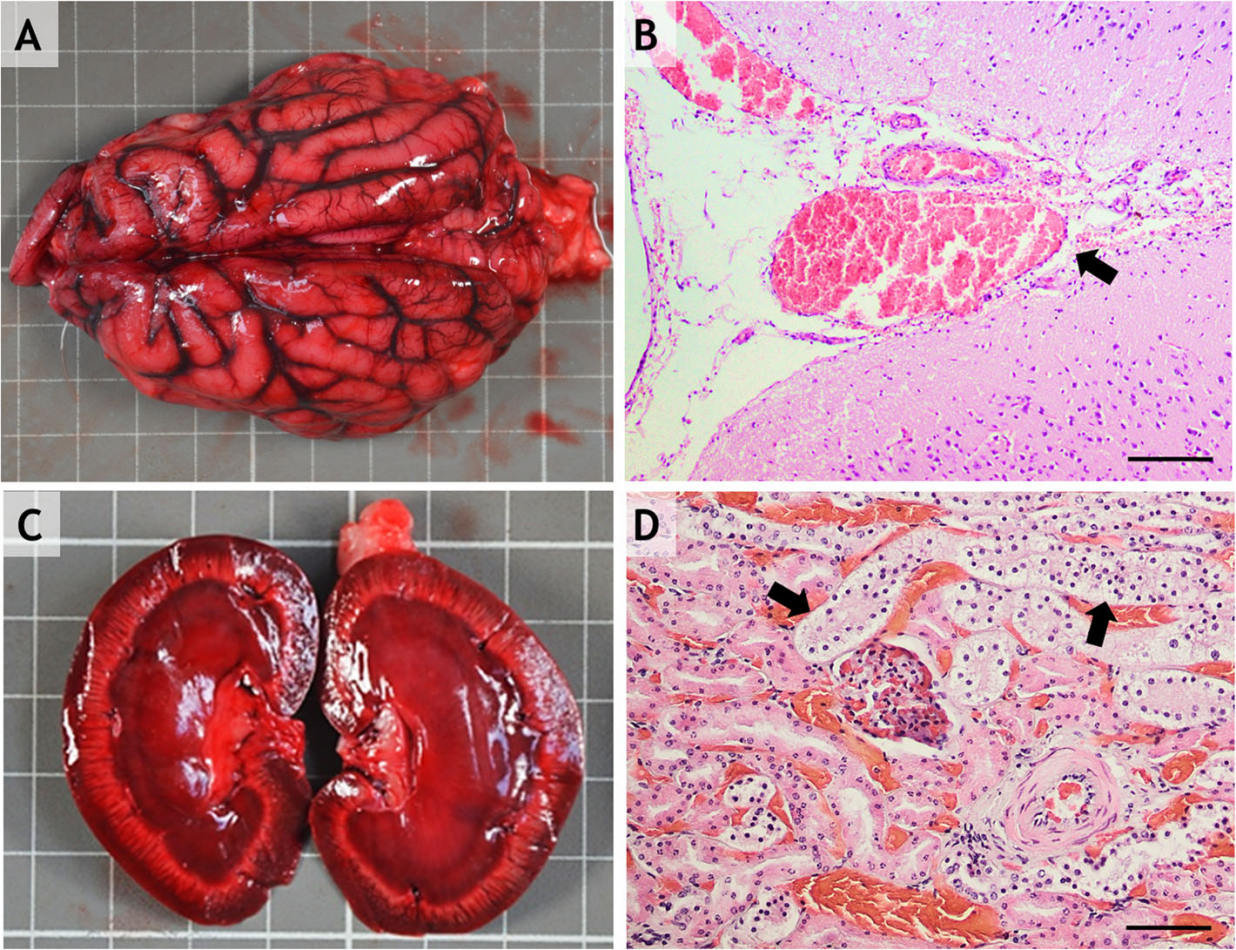
**a**: Diffuse subendocardial hemorrhages. **b**: Myocardial congestion and hemorrhage H-E staining, 10x. **c**: Diffuse gastric congestion and gastric content containing a pink foreign substance. **d**: Diffuse gastric congestion within the lamina propria and submucosa; H-E staining, 10x; scale bar = 200 μm

In the brain, the main findings were bilateral meningeal and cerebral acute congestion (Fig. 4a) with occasional petechiae. Histologically, the main changes observed in the brain were represented by cerebral congestion and gliosis, including the presence of glial nodules and discrete vascular cuffing (margination), mainly with lymphocytes. Additionally, some neurons presented a dark brown material (most likely lipofuscin) in the cytoplasm. The lepto-meningeal blood vessels presented congestion associated with local edema (Fig. 4.-b).

Bilateral, diffuse renal congestion accompanied by tubular degeneration and necrosis was recorded in all cases. Histologically, cortical-medullar congestion (Fig. 4-c), vacuolar degeneration of the epithelium of the renal cortical tubules (Fig. 4-d), congestion of the glomerular tuft and Bowman’s capsule thickening associated with the presence of a proteinaceous (hyaline) material in the urinary space were found.

## Discussion

The residential and industrial use of carbamate and organophosphate pesticides is widespread in the United States. According to the US Environmental Protection Agency in 1997, over 40 organophosphate pesticides and 22 carbamate pesticides were included in the list of 900 pesticides that posed the highest risks to human health and were registered for use in the United States [25]. Both organophosphate and carbamate pesticides primarily target the nervous system of insects. Because of the structural similarities with the physiologically-active biomolecules, carbamates are synaptic-release blockers of the acetylcholinesterase (AChE). By this competitive-inhibitor mechanism, carbamates determine the excessive synaptic accumulation of acetylcholine (ACh) and disrupt the transmission of the neurological impulse, leading to the clinical signs associated with cholinergic toxicity. Physostigmine (eserine alkaloid), and organophosphates are additional examples of reversible and respectively irreversible cholinesterase inhibitors [11, 25].

In addition to neurological deficiency, neurodegeneration, endocrine disruption, and oncogenesis, organophosphates and carbamates can induce, whether directly or indirectly, extensive toxic effects in several systems as cardiovascular, respiratory, immune, urinary, reproductive, musculoskeletal, and cutaneous [25].

Carbofuran (furadan) is still causing intoxication in animals even after almost a decade of being banned [26]. Illegal poisoning of wildlife and domestic animals is a worldwide issue [23]. There are high numbers of carbofuran poisoning incidents in birds. Novotny et al. [23] found sporadic cases of small carnivore intoxication, and martens and foxes are thought to be the main object of poisoners. Additionally, domesticated animals, such as pets, mainly dogs, and livestock, are at risk of being poisoned with carbofuran [8]. The clinical signs of accidental or intentional carbamate poisoning are nonspecific, reflecting a combination of muscarinic and nicotinic hyperstimulation [11].

Carbamates are reversible AChE inhibitors derived from carbamic acid. Carbamate causes inhibition of the activity of AChE, which is an enzyme responsible for the hydrolysis of the neurotransmitter acetylcholine in two separate components: choline and acetic acid [27, 28]. This results in an excess of acetylcholine in the synaptic cleft and prolonged binding to postsynaptic receptors [29]. AChE inhibition causes hyperstimulation of cholinergic receptors, followed by muscarinic, nicotinic and central nervous signs. AChE inhibitors may also impair endothelial function due to their toxicity to endothelial cells [30, 31] and the vascular wall [31]. The overstimulation of the somatic nervous system usually results in tremors, muscle twitches, and piloerection, as well as ataxia and paresis. Cholinergic tracts can be important to both autonomic parasympathetic and sympathetic systems but mostly influences the parasympathetic roots, transmitting impulses from the autonomic ganglia to all major organs of the body [3].

Systemic effects may occur within 30–60 min, generally occur after 6 h, and rarely occur after 12 h. Muscarinic symptoms are usually associated with salivation, lacrimation, urination, diarrhea, and gastroenteritis (SLUDGE) in addition to bradycardia, dyspnea, and miosis. Local effects usually occur because of direct contact with the product. Symptoms can be observed after a few minutes or can be delayed several days in the case of cutaneous exposure [32].

Intoxication with a cholinesterase inhibitor may lead to apparently opposite clinical signs, such as either constriction or dilation of the pupils or a speeding up or slowing down of the heartbeat.

The autonomic nervous system is subjected to constant adjustment through feedback mechanisms, and because of this, each individual may react differently to various levels of cholinergic stimulation. Death usually occurs due to respiratory failure and cardiac arrest [7].

The 7 cases described here are a clear example of carbofuran used for the intentional poisoning of dogs. In most of these confirmed cases, the results were used by authorities in legal investigations. According to police report information, there are common reasons for killing both dogs and cats, many of which are related to domestic or social violence [33–35].

The investigation of cases of intentional animal poisoning is as serious as that in human cases [36, 37], yet it is a very challenging and difficult process [15].

The inquiry of an incident that has as result the sudden death of animals generally starts with a field investigation, full gross post-mortem examination, and finally, if considered relevant for the case chemical-toxicological testing for toxin identification [38]. Multistage mass spectrometry (MS/MS) when coupled with chromatography can identify low levels of an analyte in case of pesticide poisoning [39]. Due to the previously reported high sensitivity and selectivity of the analytical determination in case of pesticide intoxication [39] a triple quadrupole mass spectrometry analyzer operated in the selective reaction monitoring mode, was used for our chemical toxicological investigation. The main differences of protocol compared with the technique optimized by Luzardo et al. [39], was the type of matrix (they used liver) and the weight (2 g of sample) of the samples, as well as the dilutions and the quantity of the solvent. Similarly, the use of sonication should be mentioned, which improves the extraction efficiency and recovery rate of certain key pesticides, such as carbofuran. Therefore, Luzardo et al. [39] added a 5-min sonication to the extraction protocol; in our cases, sonication was performed for 15 min. Another method using 2 g of homogenized liver samples is based on a new analytical multiclass method named the Quick, Easy, Cheap, Rugged and Safe (QuEChERS) technique [40], developed by Sell et al. and validated according to the requirements of SANCO/12571/2013 [41].

In our study, pathological examinations revealed predominant pulmonary lesions. Thus, carbofuran poisoning induced respiratory and cardiac depression, which led to the death of the dogs. Hyperstimulation affects vascular tone and cell permeability and tissue perfusion [42], which could cause interstitial blood pooling (congestion) and edema. Similar to Motas-Gusman et al. [43], we found acute pulmonary congestion, pulmonary edema, and emphysema but without constriction or bronchial rupture. Pulmonary hemorrhage is typically described, especially in acute intoxication cases [11], and these lesions were also present in our study. Novotny et al. [23] reported dried saliva around the oral cavity, congestion of the organs and hemorrhagic necrosis of the small gut. In our study, we found only one dog with foamy salivation; four dogs presented epistaxis and four had staining by a pink-colored foreign substance (interpreted as being the consumed carbofuran) around the oral cavity. In 6 cases, we observed ocular changes consisting of conjunctival hemorrhages or congestion and unilateral or bilateral hyphema (Table 1).

## Conclusions

Currently, although some carbamates have been banned in the European Union, carbamate poisoning is still encountered, especially in wild animals [44] In this study, we described the gross and histopathological changes present in 7 dogs with acute carbamate poisoning. The most frequently encountered changes were located within the respiratory system and were represented by diffuse tracheal congestion, pulmonary congestion, hemorrhage, and edema. Additionally, meningeal cerebral congestion and hemorrhage along with renal congestion, diffuse hepatic and splenic congestion were frequently observed. In some cases, a pink foreign substance was found on the muzzle and on the fur. According to the bait examination and toxicological results, this was interpreted as being the carbofuran-containing poison. This observation could be an indication of furadan intoxication for clinicians, especially if it is associated with suggestive clinical signs or sudden death in the animals.

## Methods

The seven cases (Table 2.) of intentional carbofuran poisoning included in this study were identified in the archives of the Pathology Department of the University of Agricultural Sciences and Veterinary Medicine Cluj-Napoca, Romania. All cases were submitted for pathological diagnosis between 2015 and 2017, and all of the cases had a registered number from the Pathology Department of the University of Agricultural Sciences and Veterinary Medicine Cluj-Napoca, Romania, where all of the data is located. In four cases, the local authority requested detailed investigations, and in the remaining three cases, the animals were submitted for necropsy by their owners.

**Table 2:** Case histories and clinical findings

Case	Age	Sex	Race	Clinical history	Baits Presence	Coloured fur
**I**	6	F	Common breed	Vomiting, muscle tremors, death in 1 h	No	Yes
**II**	~	M	Common breed	Acute death	Yes	Yes
**III**	~	M	German shephard	Acute death	Yes	No
**IV**	~	F	German shephard	Acute death	Yes	Yes
**V**	~	M	Common breed	Acute death	Yes	Yes
**VI**	4	M	Common breed	Found dead with foamy saliva	Yes	No
**VII**	1	M	Common breed	Acute death	Yes	No

This study was approved by the institutional ethics committee, the “Comisia de Bioetica”, of the University of Agricultural Sciences and Veterinary Medicine Cluj-Napoca.

In all the cases, the clinical history and the pathological and toxicological findings (including the examination of the bait) were reviewed.

The baits were found near the animals or in the gastric contents and presented a pink color; most baits consisted of a mixture of bread or different kinds of meat (sheep or chicken meat or viscera, including feathers).

A complete postmortem necropsy and histopathology examination were performed in all the cases less than 24 h after death.

For the histological examination, samples were fixed in 10% buffered neutral formalin and routinely embedded in paraffin, and 4-μm sections were prepared and stained with hematoxylin-eosin (H-E).

During the necropsy, samples of the gastric content and from the baits were collected and submitted for toxicological examination.

In this study, the toxicological examination was performed by gas chromatography coupled with triple quadrupole mass spectrometry (GC-MS/MS) using solid (bait) and liquid samples (gastric content) [26, 45]. In all the cases, the samples indicated carbofuran intoxication.

The analysis was conducted by the national reference laboratory of the National Sanitary Veterinary and Food Safety Agency of Romania in Cluj-Napoca using standard Romanian methods: SR EN 1528–2:2003 and SR EN 1528–1,3,4:2004 [45, 46] which use as reference documents: Regulation (EC) No 396/2005 of the European Parliament and of the Council of 23 February 2005 on maximum residue levels of pesticides in or on food and feed of plant and animal origin and amending Council Directive 91/414/EECText with EEA relevance; 2002/657/EC: Commission Decision of 12 August 2002 implementing Council Directive 96/23/EC concerning the performance of analytical methods and the interpretation of results and SANCO/2004/2726-rev 4-December 20,081 Guidelines for the implementation of decision 2002/657/EC 2. The validation parameters were: linearity, selectivity, limit of detection (LOD, 3 μg/kg), limit of quantitation (LOQ, 10 μg/kg), recovery, repeatability, reproducibility, stability, robustness and matrix effect.

For the toxicological exam, 5-g samples were extracted by acetone, homogenized after adding methylene chloride and ethyl acetate and centrifuged. The whole organic extract was evaporated to dryness under gentle nitrogen stream at about 40 °C with a nitrogen evaporator. Then, they were quantitatively passed through a gel-permeation purification vial, and a mixture of cyclohexane and ethyl acetate was added. The purified samples were evaporated to dryness under gentle nitrogen stream at about 40 °C in the nitrogen evaporator. Hexane was added to the residue, and the mixture was stirred, sonicated, centrifuged and then injected into the GC-MS/MS equipment [45, 46].

## Data Availability

The data that support the findings of this study are available from the Department of Veterinary Pathology, University of Agricultural Sciences and Veterinary Medicine; the cases have registration numbers. Data are, however, available from the authors upon reasonable request and with the permission of the Department of Veterinary Pathology, University of Agricultural Sciences and Veterinary Medicine.

## Abbreviations

AChE: Acetylcholinesterase
GC-MS/MS: Gas chromatography–multistage mass spectrometry
H-E: Hematoxylin-eosin
QuEChERS: Quick, Easy, Cheap, Rugged and Safe
LOD: Limit of detection
LOQ: Limit of quantitation
